# Expected change: a new concept for monitoring patients on oral bisphosphonates

**DOI:** 10.1093/jbmr/zjag039

**Published:** 2026-02-23

**Authors:** Marian Schini, Margaret A Paggiosi, Fatma Gossiel, Steven R Cummings, Richard Eastell

**Affiliations:** Division of Clinical Medicine, School of Medicine and Population Health, University of Sheffield, Sheffield S10 2RX, United Kingdom; Division of Clinical Medicine, School of Medicine and Population Health, University of Sheffield, Sheffield S10 2RX, United Kingdom; Division of Clinical Medicine, School of Medicine and Population Health, University of Sheffield, Sheffield S10 2RX, United Kingdom; San Francisco Coordinating Center, CA 94158, United States; Division of Clinical Medicine, School of Medicine and Population Health, University of Sheffield, Sheffield S10 2RX, United Kingdom

**Keywords:** bone turnover markers, osteoporosis, treatment, bisphosphonates

## Abstract

It is essential to closely monitor the response to oral bisphosphonate therapy for osteoporosis, as many patients are nonadherent. The conventional approach is to monitor whether changes in BMD or bone turnover markers (BTMs) exceed the least significant change. This approach assumed that if a patient were not receiving a treatment, there would be no change in the BMD. We propose an alternative approach, that of expected change. We define this expected change as the change in BMD (at 24 mo) or BTMs (at 3 mo) that is exceeded in 90% of patients who are adherent with oral bisphosphonate therapy. We studied 108 postmenopausal women (age < 85 yr) who were randomized to the licensed dose of alendronate, ibandronate, or risedronate treatment for 2 yr, along with calcium and vitamin D supplementation. We identified the performance of BMD and BTMs in 3 ways. We calculated the signal-to-noise ratio, which was lower for BMD (4.1 and 2.1 for lumbar spine BMD (LSBMD) and total hip BMD (THBMD), respectively) compared to BTMs (9.4 and 10.2 for C-telopeptide of type I collagen (CTX) and procollagen I N-propeptide (PINP), respectively). We estimated the response rate as the percentage of women exceeding the least significant change, which was lower for BMD (47% and 24% for LSBMD and THBMD, respectively) than for BTMs (96% and 94% for CTX and PINP, respectively). We estimated the expected change as the 90th (or 10th) percentile of change in adherent patients. We required the expected change to exceed the least significant change, and this was not observed for LSBMD and THBMD, but it was observed for CTX and PINP (expected changes of 0.233 and 12.1 ng/mL, respectively). Thus, the BTMs CTX and PINP showed the best performance as response markers for monitoring oral bisphosphonate treatment, and the new approach is based on a biological rather than a statistical endpoint when using the expected change approach.

## Introduction

The oral administration of bisphosphonates is the most common way to treat postmenopausal osteoporosis. However, the major problem with this treatment is poor adherence. More than half of women do not persist with the treatment for more than 12 mo. Since the introduction of DXA in 1987, BMD has been the primary method for identifying treatment response. However, the increase in BMD with oral bisphosphonates takes up to 2 yr to reach maximal response, and the change is small compared to the variability in the measurement. In contrast, bone turnover markers (BTMs) decrease maximally within 3 mo of starting treatment and exhibit a significant change relative to their inherent variability. Thus, the International Osteoporosis Foundation and the European Calcified Tissue Society recommended the use of a bone resorption marker (C-telopeptide of type I collagen, CTX) and a bone formation marker (procollagen I N-propeptide, PINP) to identify poor adherence in patients taking oral bisphosphonates.^1^

The statistical concept of least significant change^2^ is applied to the change in measurements made at baseline and 3 mo. A change greater than the least significant change (LSC) provides 95% CI that the observed change exceeds the physiological variation for that marker and suggests a clinically significant change.^3^

One drawback of using LSC is that it is a statistical method. Least significant change can be reduced by making multiple measurements. For example, by taking 8 measurements on each occasion instead of 1, you reduce the threshold for a “significant change” from 2.77 times the SD to 0.98 times the SD. This issue has nothing to do with whether the person is responding, that is, it is not a biological method of monitoring. Moreover, LSC is calculated for people not taking treatment.

We are aware of the biological responses of BMD and BTMs, as reported in clinical trial publications. The usual approach is to give the mean change and its 95% CI. For example, after 3 yr, the mean change (and 95% CI) for LS-BMD and CTX after treatment of postmenopausal osteoporosis with zoledronate 5 mg annually is 6.7% (5.5%-7.7%) and 59% (55%-63%), respectively.^4^ However, CIs are for populations, not for individuals. We need to know the prediction limits, which would allow us to determine whether the response is what is expected for a person who adheres to the medication.

The study aims to identify the best biomarker using three approaches. First, to relate the response of the marker (change) to the variability [coefficient of variation^5^ or root mean square deviation (RMS SD)] in a signal-to-noise ratio. Second, the study utilizes the change beyond the LSC as a definition of response, comparing the response rate of BMD at 24 mo and BTMs at 3 mo. Third, a promising biomarker would have a minimal expected change (90th percentile) that is greater than the LSC; otherwise, we would have the paradox of no treatment response in a person with the expected change. Thus, we will examine whether the minimal expected change exceeds the LSC in postmenopausal osteoporosis treated with alendronate, ibandronate, or risedronate.

A further aim of the study is to investigate the proportion of people who are partially adherent who respond.

## Materials and methods

### Study design

The TRIO study was a 2-yr, open-label, parallel, randomized control intervention trial of three oral bisphosphonates. The design was previously published.^6^ In brief, postmenopausal women with osteoporosis were recruited [BMD at the LS or proximal femur of (1) T-score ≤ −2.5 or (2) T-score ≤ −1.0] plus a prevalent nontraumatic fracture. Inclusion criteria: these were all ambulatory women, less than 85 yr old, more than 5 yr postmenopausal and able to give informed consent. Exclusion criteria were fracture in the previous 12 mo, the use of medications or diagnosis of any disease or medical condition known to affect bone or a BMI outside the range of 18-35 kg/m^2^. The participants were randomized to receive 1 of 3 oral bisphosphonates at the licensed dose: (1) ibandronate (Bonviva, Roche, 150 mg once a month), (2) alendronate (Fosamax, Merck, 70 mg once a week), or (3) risedronate (Actonel, Warner Chilcott, 35 mg once a week). Adherence was assessed electronically using medical events monitoring system bottle caps^7^(AARDEX). All women received calcium carbonate 3 g (1200 mg elemental calcium) and cholecalciferol 20 μg (800 IU) per day (Adcal D3, 2 tablets daily, ProStrakan); these were initiated 1 wk before the bisphosphonate (Baseline 1 visit).^6^

BMD (g/cm^2^) was measured by DXA using a Discovery A densitometer (Hologic Inc.). Blood and urine tests were performed at baseline 1 (week 1), baseline 2^8^ then at 1, 2, 4, 12, 13, 48, and 96 wk. Blood was collected after an overnight fast, and the sample was left to clot for 30 min at room temperature before centrifugation at 2500 × *g* for 10 min. Second void fasting morning urine samples were collected. Samples were stored at −80 °C until analysis, and all visits of individual participants were measured in one analytical batch. The CTX, N-mid osteocalcin (OC), intact PINP, and bone alkaline phosphatase (BALP) were measured using the IDS-iSYS automated immunoassays (Immunodiagnostic Systems). The inter-assay coefficients of variation (CVs) were 6.5%, 5.0%, 7.2%, and 3.5%, respectively. The N-telopeptide of type I collagen (NTX) was measured in urine by an automated competitive immunoassay (Vitros ECi, Ortho-Clinical Diagnostics; inter-assay CV 6%). The NTX was expressed as a ratio to urinary creatinine concentration measured by the dry slide method (Vitros 250, Ortho-Clinical Diagnostics; inter-assay CV 3%).^9^

### Statistical analysis

We first used the MEMS data to assess adherence with treatment at 48 wk. Adherence was defined as the patient having taken more than 80% of the tablets issued; this definition of adequate adherence was used in a previous study using TRIO^6^ and has been justified by a literature review.^10^ BTMs and BMD were measured at week 12 and 13, and we used this data to calculate the LSC following the International Society for Clinical Densitometry (ISCD) principles for duplicate samples.^11^

In brief, we first calculated the within-subject variance for each subject using the repeated measures (ie, data from week 12 and 13); the difference between the 2 measurements (x_1_, x_2_) was squared, then divided by 2.


$${\mathrm{Var}}_{\mathrm{subject}}={\left({\mathrm{x}}_1-{\mathrm{x}}_2\right)}^2/2.$$

Then, we calculated the following parameters.

Root mean square (RMS): The square root of the average within-subject variance across all subjects.

Coefficient of variation (CV%): Calculated by dividing the RMS by the pooled mean of the biomarker values, then multiplying by 100.

Least significant change (95% LSC): The absolute LSC was calculated by multiplying the RMS by 2.77. The percent LSC was calculated by multiplying the CV% by 2.77 (1.96 × sq root of 2, the value 1.96 is the z-score that corresponds to 95% CI in the standard normal distribution).

To calculate the expected change, we used the mean value of the BTMs at weeks 12 and 13. We chose this time point because we expected to see a change in BTMs by then. We named this value the month 3 measurement. We then calculated the difference between month 3 and Baseline 1 for BTMs (absolute change) and the percent change for BMD between week 96 and screening. The 10th and 90th percentiles of the change were arbitrarily designated defining response. In terms of BTMs, as we would expect a decrease with treatment, we named the value at the 90th percentile—the minimal expected change that is the minimal change in BTMs that we would expect in 90% of adherent patients. For BMD, we expected an increase, and we used the value at the 10th percentile, which we termed the minimal expected change, as this is the minimal change that we would expect in 90% of adherent patients. To test whether those thresholds identified distinct populations of nonresponders, we checked whether the distribution for these markers (for all treatments) was bimodal; for that, we used Hartigan’s dip test for unimodality/multimodality.

To ensure a patient is responding, we would expect the minimal expected change to exceed the LSC. Therefore, we checked which BTMs or BMDs followed this criterion. We used the LSC for all bisphosphonates in this study to allow for a larger sample size.

We also wanted to check the signal-to-noise ratio (SNR) to check which markers perform better for monitoring.

The SNR for BTM = mean absolute change at month 3/RMS.

SNR for BMD = mean percent change at 96 wk/CV%.

For the further aim of the study, we defined as nonadherent the patients having taken less than 50% of the tablets issued. We used the promising markers from the first part of the study and checked how they performed in these patients. We used the minimal expected change calculated from the first part of the study and tested these patients against these thresholds.

All the analyses were done at R Studio (RStudio 2022.12.0+353 “Elsbeth Geranium” Release, 2009-2022 Posit Software, PBC).

## Results

### Adherent patients

In total, 108 women participating in the TRIO study were treatment-adherent (*n* = 37 on alendronate, *n* = 32 on risedronate, and *n* = 39 on ibandronate). The baseline characteristics are shown in Table 1.

**Table 1:** Baseline characteristics of the adherent participants.

	**Adherent (*n* = 108)**		**Nonadherent (*n* = 11)**	
**Variable**	**Mean**	**SD**	**Mean**	**SD**
**Height, cm**	160.6	5.5	159.7	5.6
**Weight, kg**	67.2	10.2	74.8	9.3
**THBMD, g/cm** ^**2**^	0.77	0.10	0.84	0.08
**LSBMD, g/cm** ^**2**^	0.79	0.11	0.83	0.08
**FNBMD, g/cm** ^**2**^	0.65	0.10	0.71	0.09
**PTH, ng/L**	38.1	12.9	39.5	9.9
**Vitamin D, ng/mL**	26.0	10.5	24.5	10.1
**CTX, ng/mL**	0.650	0.270	0.635	0.225
**PINP, ng/mL**	49.1	16.9	42.4	11.8
**Osteocalcin, ng/mL**	27.7	10.1	22.2	5.4
**BALP, ng/mL**	20.7	7.9	21.4	5.7
**NTX, nmol/mmol Cr**	48.0	21.0	62.3	68.2

Abbreviations: BALP, bone alkaline phosphatase, CTX, C-telopeptide of type I collagen; FNBMD, femoral neck BMD; LSBMD, lumbar spine BMD; NTX, N-telopeptide of type I collagen; PINP, pro-collagen I N-propeptide; PTH, parathyroid hormone^12^; SD, standard deviation; total hip BMD (THBMD).

In Table 2, we present the minimal expected change for BTMs at month 3 (in absolute units) and in BMD at 96 wk (in percent change). For example, PINP decreased by 12.1 ng/mL in 90% of patients while LSBMD increased by 1.93%. PINP, CTX, and OC are the markers in which the expected change exceeds the LSC (Table 2).

**Table 2:** Comparison of expected change and LSC in all adherent patients in all treatment groups (*n* = 108).

**All treatment groups**			
**Measurement**	**Minimal expected change**	**LSC (absolute for BTM, % for BMD)**	**Expected change > LSC**
**Bone turnover markers at 3 mo**			
**PINP, ng/mL**	−12.1	−8.11	yes
**CTX, ng/mL**	−0.233	−0.146	yes
**BALP, ng/mL**	−1.46	−3.94	no
**Osteocalcin, ng/mL**	−3.64	−4.06	no
**NTX/Cr, nmol/mmol Cr**	−5.13	−27.4	no
**BMD at 96 wk**			
**Lumbar spine %**	1.930	4.403	no
**Total hip %**	−0.493	4.074	no
**Femoral neck %**	−0.700	8.421	no

Abbreviations: BALP, bone alkaline phosphatase; BTM, bone turnover markers; CTX, C-telopeptide of type I collagen; FNBMD, femoral neck BMD; LSBMD, lumbar spine BMD; LSC, least significant change^2^; NTX, N-telopeptide of type I collagen; PINP, pro-collagen I N-propeptide; THBMD, total hip BMD.

In Table 2, we note that for both the FN and TH the expected change can be negative. The explanation for the negative change could be the smaller decreases in BMD. The correlation between change in total hip BMD (THBMD) and BTMs was significant (PINP *p* = .04, r = −0.24, CTX *p* = .03, and r = −0.25).

Table 3 shows data on responders and SNR. PINP, CTX, and OC have higher SNRs than the other parameters studied. Table 3 shows the number of responders per LSC. Once again, it appears that BTMs are outperforming BMD. We found a significant correlation between changes in CTX and PINP at month 3 (r = 0.6, *p* < .0001) (Figure 2). Figure 1 shows violin plots of the change in BTMs proposed by the International Osteoporosis Foundation (IOF) and European Calcified Tissue Society (ECTS) and for LS and THBMD.

**Figure 1:**
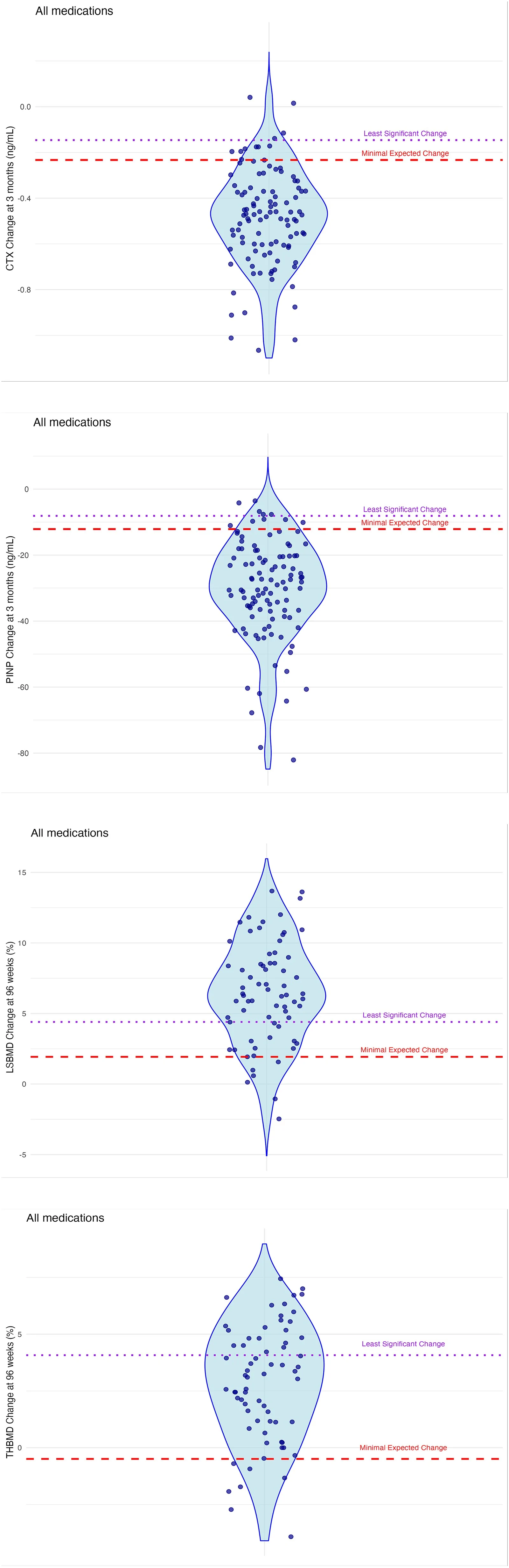
Violin plots of the change in BTM (absolute) and BMD (percent). The graphs show data on CTX (row 1), PINP (row 2), LSBMD (row 3), and THBMD (row 4). The red dashed horizontal line shows the minimal expected change and the purple dotted line the least significant change. With BTMs, we expect a decrease from baseline after bisphosphonate treatment, while with BMD, we expect an increase. A violin plot displays the full distribution of the data. The width of the “violin” at each value on the y-axis represents how frequently observations occur at that level (a smoothed density estimate). Wider sections indicate more common values, while narrower sections indicate less common values. Individual data points are shown as jittered dots to illustrate the underlying sample. C-telopeptide of type I collagen (CTX), pro-collagen I N-propeptide (PINP), lumbar spine BMD (LSBMD), and total hip BMD (THBMD).

**Table 3:** Comparison of signal to noise ratio and responder rates for different measurements in all adherent patients on all treatments. Mean change: at month 3 for BTM, at week 96 for BMD).

**All treatment groups**							
**Measurement**	**Mean change**	**RMS**	**CV%**	**Signal-to-noise**	**Responders by LSC (*n*)**	**Total *N***	**Responders by LSC (% of total)**
**Bone turnover markers at 3 mo**							
**PINP, ng/mL**	−29.8	2.93		−10.2	101	107	94.4
**CTX, ng/mL**	−0.498	0.0527		−9.4	103	107	96.3
**BALP, ng/mL**	−7.76	1.42		−5.5	78	107	72.9
**Osteocalcin, ng/mL**	−10.6	1.46		−7.3	91	106	85.8
**NTX/Cr, nmol/mmol Cr**	−28.4	9.89		−2.9	47	106	44.3
**BMD at 96 wk**							
**Lumbar spine %**	6.45		1.59	4.1	51	71	47.2
**Total hip %**	3.05		1.47	2.1	25	70	24.3
**Femoral neck %**	2.97		3.04	1.0	1	70	6.5

Abbreviations: BALP, bone alkaline phosphatase; BTM, bone turnover markers; CTX, C-telopeptide of type I collagen; CV%, coefficient of variation^5^; FNBMD, femoral neck BMD; LSBMD, lumbar spine BMD; LSC, least significant change^2^; NTX, N-telopeptide of type I collagen; PINP, pro-collagen I N-propeptide; RMS, root mean square; THBMD, total hip BMD.

### Non-adherent patients

In general, adherent patients had similar characteristics to the nonadherent patients (*n* = 11), although non adherent patients tended to be heavier and with higher baseline THBMD (Table 1). Adherent patients had better responses in both markers (mean decrease in PINP in adherent 30 and 0.50 for CTX vs 19 and 0.33 ng/mL, respectively, for nonadherent).

However, 6 of the non-adherent patients (55%) had reductions in CTX and PINP, 2 for CTX alone and 1 for PINP alone (Figure 2).

**Figure 2:**
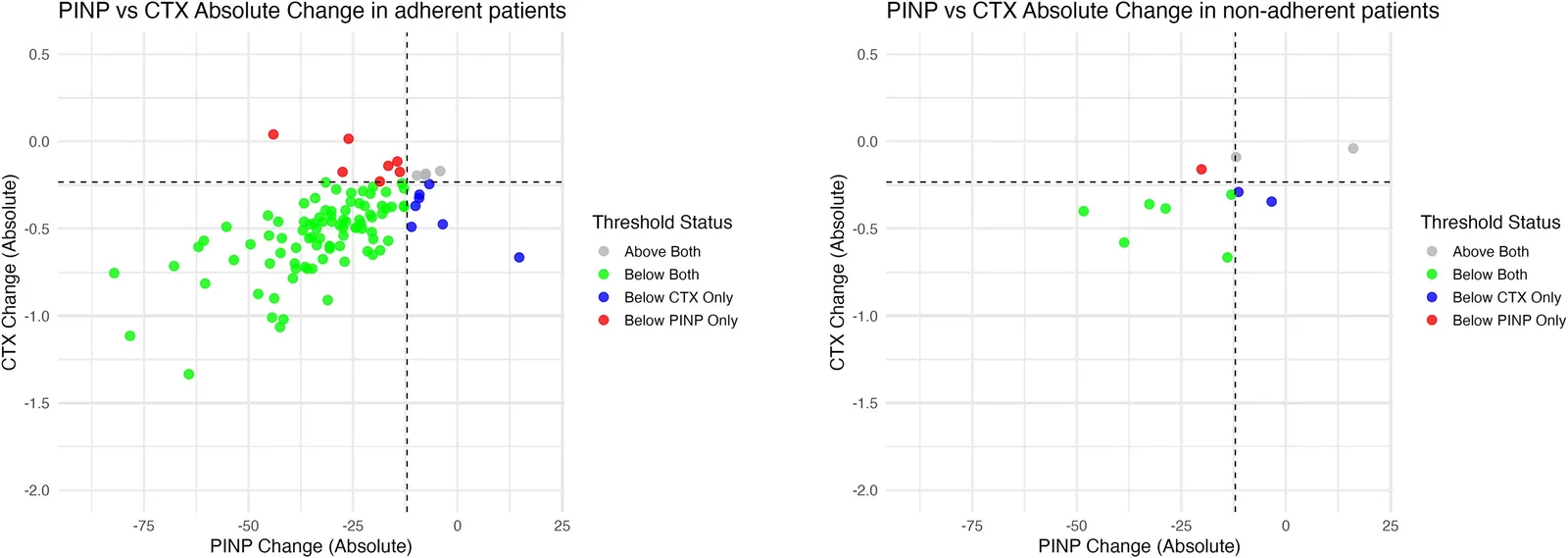
Graph showing the responders for CTX and PINP (left for adherent patients, right for non-adherent). The dotted lines represent the expected change. Green patients respond to both markers while gray ones are non-responders to both. C-telopeptide of type I collagen (CTX) and pro-collagen I N-propeptide (PINP).

## Discussion

In this study, we introduce a new concept for monitoring patients undergoing treatment for osteoporosis: the expected change. To find this, we extracted data from the TRIO study on oral bisphosphonates (alendronate, risedronate, and ibandronate) and analyzed it among women who were 80% adherent with treatment at 48 wk. The expected change concept is more clinically relevant than the current method of least significant change. We were also keen to see which biomarker (BTMs or BMD) would perform better. To achieve this, we employed 3 methods: the signal-to-noise ratio, the response rate, and whether the expected change in the biomarker studied exceeded the LSC. The conclusion is that CTX and PINP, the 2 BTMs proposed by the IOF and ECTS,^1^ are the most suitable for use and the changes in their levels correlate well. The signal-to-noise ratio concept has been used previously, and the results were quite similar, that is, BTMs are better than BMD.^13^

Seeing that the distribution of the markers PINP and CTX was not bimodal, we felt more confident that all the patients were responding.

The analysis for PINP and CTX allows for the separation of adherent patients (>80%) from those not taking any medication, as the expected change does not overlap with the least significant change. The analyses do not allow for the separation of patients who are adherent (>80%) and those who are poorly adherent (<50%). The PINP and CTX changes in the latter are less than with adherent patients, but in many cases would be classified as meeting the expected change. Our understanding of the relationship between adherence and changes in bone resorption markers can explain this finding. Thus, the effect of <50% adherence with risedronate was associated with a response of >50% in bone resorption markers CTX in more than 40% women.^14^ Additionally, the dose-response relationship for alendronate is non-linear. For example, alendronate at doses of 5 and 20 mg daily (the licensed dose was 10 mg for most indications) resulted in similar decreases in the bone resorption markers deoxypyridinoline after 6 wk of treatment.^15^ This observation of nonadherent patients appearing to respond surprised us. This paper aimed to find a better way of monitoring rather than using LSC, for the reasons already mentioned, but we do acknowledge that no method is perfect. Also, it is important for the clinician to keep in mind, that even patients who do not take all their doses might still respond. However, the smaller decrease in BTMs that we see in these patients, might be related to smaller reduction in vertebral fracture risk.^16^

The values that we gave are the absolute change in BTMs and the percentage change in BMD. In terms of BMD, percentage change has been used since the introduction of DXA devices in the 1980’s, and we can’t expect physicians to change their habits and use absolute units. For BTM, it is easier in clinical practice to have the absolute value, as it is simpler to calculate than the percent change. Moreover, it is better to use for the offset of effect, as percent changes would be very high as the denominator is very low.

We were surprised to see negative percentage changes in TH and FN BMD. This may be because, if these patients were not treated, the decrease in BMD would be greater than the one we see with treatment. Also, we noted a negative correlation between changes in hip BMD and BTMs.

There are limitations to using the EC approach. Namely, the expected change is treatment-specific. If we studied a potent drug, such as denosumab, we would see much bigger expected changes, and if we studied a less potent drug, such as raloxifene, we would see much smaller expected changes (and even lower than LSC) than we have reported for oral bisphosphonates. We wanted to study oral bisphosphonates, as they are the most common drugs used for osteoporosis in most countries. In the USA, alendronate alone makes up 65% of prescriptions.^13^ Moreover, we acknowledge that the TRIO study included only postmenopausal women, so results might differ in men. We also recognize the small number of participants.

Furthermore, all serial measurements are subject to the regression-to-the-mean artifact; therefore, in patients who fail to respond at the first measurement, a subsequent measurement may be helpful. This could be an example of a false negative; a false positive is also possible, but difficult to detect. Another statistical issue is that of serial correlation. We evaluate variability over 1 wk; had we used a longer period, the variability would have been greater. However, this would apply equally to all the markers we were comparing.

We also think that by doing the analysis at 3 mo, we might not have seen the full decrease expected in PINP. So, in our suggestions below, we recommend monitoring at 4-6 mo.

Another limitation is that we did not have a placebo group and results might be difficult to interpret. In a previous study of ours which established the LSC from trials of teriparatide, we obtained the variability from a placebo group.^17^ However, a placebo group might not be such a good idea, as participants in placebo groups are usually on calcium supplements, which can reduce BTMs, especially CTX. We have shown that a small percentage of patients with osteoporosis have PINP less than 35 ng/mL (less than 25% in the TRIO study), and these do not exceed the LSC, nor would they be likely to meet the expected change.^18^ Lastly, for our non-adherent analysis, the change in BTMs was at 3 mo, while adherence was at 48 wk. So, a patient may have been adherent at first when the BTMs were measured, but not by week 48. There are studies that show that adherence decreases with time.^10^ In addition, if you estimate the expected change at 6 mo, it may be different and the percentage of “good response” in the non-adherent patients may decrease.

To illustrate the use of expected change, let’s consider a patient starting an oral bisphosphonate with a baseline PINP of 45 ng/mL. The expected change for PINP is −12.1 ng/mL. So, if the change at 4-6 mo is more than this value, then we can tell them with an 90% certainty, that they are having the change we expect for them on treatment with oral bisphosphonates (Figure 3). If the patient is not responding, adherence should be checked. Moreover, the clinician should make sure that there has not been a fracture or a new disease that could be affecting BTMs. Parenteral treatment should be considered if despite adherence and no fracture or disease, the patient seems not to be responding. If a baseline value is unavailable, the premenopausal median approach can be used (decrease of PINP to values below 35 ng/mL and of CTX 0.280 ng/mL.^3^

**Figure 3:**
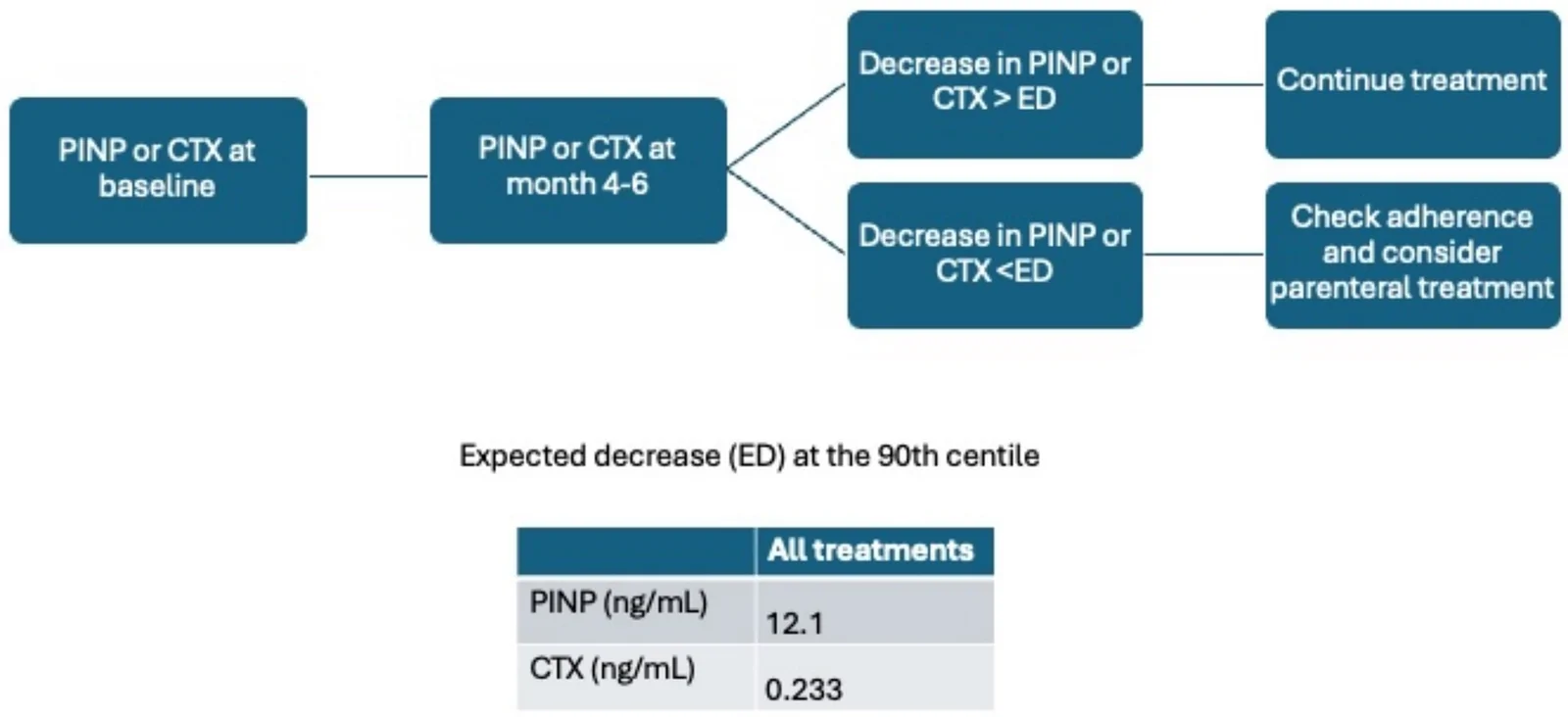
Flow chart for proposed patient monitoring.

At this point, we need to note that part of our previous work focused on promoting the use of THBMD as a surrogate marker for clinical trials in osteoporosis (SABRE study).^19^ However, in this project, BMD is not a good way for monitoring. The reason for this discrepancy is that the SABRE project is designed for clinical trials, not for individual patients. Moreover, BMD is a surrogate for antifracture efficacy in clinical trials, while BTMs are markers for monitoring adherence and response to treatment. In SABRE, BTMs performed well, particularly for vertebral fractures, but fewer results are available.^16^

In conclusion, expected change could be considered in clinical practice for monitoring patients on osteoporosis treatment, in order to identify response to the treatment but does not give information on the impact on fracture risk. In our view, this is better than using the LSC which is a statistical method which comes for people not on treatment. Our data support the use of PINP and CTX for treatment monitoring of oral bisphosphonates. We understand that this data is preliminary, and we plan to study the same approach to other drugs, such as denosumab and teriparatide. Our results should be confirmed in larger phase 3 studies that include oral bisphosphonates. These studies should be able to demonstrate good adherence.

## Data Availability

Data available on request.
